# Sodium alginate microencapsulation improves the short-term oral bioavailability of cannabidiol when administered with deoxycholic acid

**DOI:** 10.1371/journal.pone.0243858

**Published:** 2021-06-17

**Authors:** Maimuna Majimbi, Emily Brook, Peter Galettis, Edward Eden, Hani Al-Salami, Armin Mooranian, Hesham Al-Sallami, Virginie Lam, John C. L. Mamo, Ryusuke Takechi

**Affiliations:** 1 Curtin Health Innovation Research Institute, Curtin University, Perth, WA, Australia; 2 School of Population Health, Faculty of Health Sciences, Curtin University, Perth, WA, Australia; 3 Curtin Medical School, Faculty of Health Sciences, Curtin University, Perth, WA, Australia; 4 School of Medicine and Public Health, University of Newcastle and The Australian Centre for Cannabinoid Clinical and Research Excellence, Newcastle, NSW, Australia; 5 School of Pharmacy, University of Otago, Otago, New Zealand; St. John’s University College of Pharmacy and Health Sciences, UNITED STATES

## Abstract

**Background:**

Cannabidiol (CBD) confers therapeutic effects in some neurological disorders via modulation of inflammatory, oxidative and cell-signalling pathways. However, CBD is lipophilic and highly photooxidative with low oral bioavailability in plasma and brain. In this study, we aimed to design and test a CBD microencapsulation method as a drug delivery strategy to improve the absorption of CBD. Additionally, we evaluated the brain uptake of CBD capsules when administered alongside capsules containing a permeation-modifying bile acid, deoxycholic acid (DCA).

**Methods:**

Microcapsules containing either CBD or DCA were formed using the ionic gelation method with 1.5% sodium alginate formulations and 100 mM calcium chloride. C57BL/6J wild type mice randomly assigned to three treatment groups (3–4 mice per group) were administered CBD in the following preparations: 1) CBD capsules, 2) CBD capsules + DCA capsules and 3) naked CBD oil (control). To assess the short-term bioavailability of CBD, plasma and brain samples were collected at 0.3, 1 and 3 hours post administration and CBD levels were analysed with liquid chromatography mass spectrometer.

**Results:**

We produced spherical capsules at 400 ± 50 μm in size. The CBD capsules were calculated to have a drug loading of 2% and an encapsulation efficiency of 23%. Mice that received CBD capsules + DCA capsules showed a 40% and 47% increase in CBD plasma concentration compared to mice on CBD capsules and naked CBD oil, respectively. Furthermore, the CBD capsules + DCA capsules group showed a 48% and 25% increase in CBD brain concentration compared to mice on CBD capsules and naked CBD oil, respectively. In mice treated with CBD capsules + DCA capsules, the brain CBD concentration peaked at 0.3 hours with a 300% increased availability compared to CBD capsules and naked CBD oil groups, which peaked at 1 hour after administration.

**Conclusions:**

The microencapsulation method combined with a permeation enhancer, DCA increased the short-term bioavailability of CBD in plasma and brain.

## Introduction

Cannabidiol (CBD) is a potent non-psychoactive constituent in marijuana (*Cannabis sativa)* with no reported intoxicating effects unlike tetrahydrocannabinol (THC) [1]. CBD binds to receptors CB1, CB2 and 5HT1A to modulate cellular activity and inhibit excitotoxicity [2]. The anti-inflammatory and antioxidant properties of CBD have been reported across numerous pathologies, including neurodegenerative and metabolic diseases. Studies show that repeated administration of CBD may be neuroprotective in animal models of Alzheimer’s disease via decreasing microglial activation and attenuation of memory deficits [3]. Furthermore, activation of the endocannabinoid system has been shown to preserve the cerebral capillary endothelium that forms the blood brain barrier (BBB) [4, 5] and exert therapeutic effects in animal models of diabetes [6].

CBD is highly lipophilic, sensitive to light and largely broken down in the duodenum resulting in extremely low oral bioavailability in plasma and tissues (approximately 6% and 1%, respectively) [7]. Whilst chronic usage of CBD is clinically well tolerated, its lability poses limitations for use in research and adaptation as a pharmacotherapy [8]. Therefore, emerging studies have focused on encapsulation techniques that function as a vehicle for CBD [9].

Sodium alginate is an extract from brown algae that is biocompatible, hydrophilic, non-toxic and readily available for human use. The alginate cross-links with multivalent ions like Ca^2+^ to form stable hydrogel polymers under mild aqueous conditions [10]. Thus, calcium alginate microcapsules provide a ‘physical’ barrier for volatile drugs such as CBD against exposure to light and air in pharmacological protocols. Previously, our studies have demonstrated that sodium alginate microcapsules significantly increase the brain uptake and associated neuroprotective effects of probucol—the highly lipophilic, antilipidemic drug [11]. Subsequent studies of ours confirmed that microencapsulation improves therapeutic efficiency of lipophilic drugs by protecting against degradation due to low stomach pH and efflux protein activity in the intestinal epithelium [12, 13].

The use of absorption enhancers is yet another method of improving bioavailability in drug delivery research. Deoxycholic acid (DCA) is a metabolite of chenodeoxycholic acid, a primary bile acid made from cholesterol in the liver in humans [14]. Bile acids have amphiphilic properties and primarily function as surfactants in the body. DCA is among several secondary bile acids to be utilised in pharmacokinetic research as a permeation-modifying biomolecule [15]. Specifically, lipophilic drugs with low intestinal dissolution rates have shown improved absorption when administered with DCA [16]. Bile acids promote aqueous solubility and increase fluidity of phospholipid membranes, making them ideal candidates for BBB permeability experiments [16]. A report from our lab showed that co-encapsulation with a DCA variant improved the targeted-delivery effects of probucol [12]. However, there is a relative paucity of studies focusing on the permeation effects of DCA on CBD absorption. The fundamental challenges of administering DCA alongside a labile drug such as CBD with minimal exposure to the environment and impost on experimental animals have yet to be resolved.

In this study, we utilised the established sodium alginate microencapsulation method to develop CBD capsules and assess the abundance of CBD in plasma and brain samples of wild-type mice. Furthermore, we administered CBD capsules alongside DCA capsules to examine the blood-to-brain kinetics of CBD in conjunction with a permeation enhancer. The study captured CBD absorption at three time points leading up to- and immediately post its peak concentration to elucidate the short-term effects of our drug excipients. The findings of this study may provide a potential avenue for improving the penetrance of CBD in models of neurodegeneration.

## Materials and methods

### Materials

CBD (14.5% solubilised extract in Miglyol 812N, a medium chain triglyceride carrier oil) and Miglyol 812N were kindly gifted by Zelira Therapeutics, (Perth, Western Australia). Medium Viscosity Sodium Alginate (MVSA) (≥2,000 cP, 2% (25°C)), deoxycholic acid (DCA) and Calcium Chloride anhydrous (98%) were purchased from Sigma-Aldrich, (St Louis, MO, USA). Formulations were made up in HPLC grade deionised water.

### CBD and DCA formulations

Solutions containing 1.5% MVSA in 80 mL of HPLC-grade deionised water were mixed overnight. The CBD formulation was made by adding 800 μL of Miglyol 812N containing 109.24 mg CBD to the MVSA solution while protected from air and light and mixed for 3 days. The DCA formulation was made by adding 10 mg of DCA to a separate MVSA solution and mixed overnight. The MVSA solution and drug formulations were mixed at the same speed at room temperature. The formulations were prepared according to a protocol developed in-house and published previously [17, 18]. The aforementioned protocol has been optimised through a series of experiments for rheology, fluid dynamics and colloidal dispersion electrokinetic (Zeta potentials) to ensure total emulsification of the drug content into the MVSA solution.

### CBD and DCA encapsulation

The CBD and DCA formulations were encapsulated immediately after emulsification using the gelation technique with the vibrating Encapsulator B-390 (BUCHI Labortechnik, Switzerland). The encapsulation protocol was adapted from previous studies in our group [11], with minor adjustment of the capsulation conditions: frequency range of 2000 Hz and air pressure at 950 mbar through a 200 μm nozzle with a flow regulating valve set at 2 rotations from the tightest starting point.

Prepared formulations were projected into the 100 mM CaCl_2_ hardening bath, which stirred with a mild vortex, at a flow rate of 5 mL/minute and formed spherical microcapsule beads. After 10 minutes in CaCl_2_, microcapsules were sieved, rinsed with deionised water and dried with a paper towel patted under the strainer. They were placed on a petri dish, covered and dried completely at 37°C for 2.5 days. Microcapsules were analysed and used for experimentation within 48 hours of drying.

### Encapsulation efficiency

The CBD loading and capsule size were determined by using HPLC Prominence (Shimadzu LC-20AT liquid chromatographer, SIL-20A autosampler and SPD-20A-UV/Vis detector (Japan)) and particle analyser (Zetasizer 3000 HS and Mastersizer 2000, Malvern Instruments, Malvern, UK), respectively according to an established HPLC protocol from our lab [12] with some modifications to account for the known effects of oil on microencapsulation [19]. Briefly, 5 mg of microcapsules were agitated and suspended in PBS (pH 8.5) for 20 h at room temperature and centrifuged for 15 min at 13200 rpm at 10°C. The supernatant was collected and diluted with Mobile Phase Mixture (ACN:Water = 75:25). Encapsulation efficiency was determined with published formulae summarised below as the relationship between theoretical and confirmed CBD loading within the microcapsules [13].


$$Drug\;loading\;(\%)=\frac{\;CBD\;in\;microcapsules\;(mg)}{Weight\;of\;microcapsules\;(mg)}\times 100$$

$$Encapsulation\;efficiency\;(\%)=\frac{Drug\;content}{Theoretical\;content\;}\times 100$$

### *In vivo* animal experiments

Healthy C57BL/6J wild type mice obtained from Animal Resources Centre, WA were group housed in a temperature-controlled laboratory on a 12-hr light/dark cycle with standard chow and water provided *ad libitum* (14 months, female, average weight 36±3 g, n = 45). The mice were fasted overnight prior to CBD administration. Experiments were conducted according to approved animal ethics protocol (Curtin Animal Ethics Committee, approval no. ARE 2018–19).

Mice were restrained and orally administered 5 mg/kg weight CBD in oil by itself (naked CBD oil), in capsulation (CBD capsule), or in capsulation with additional 4 mg/kg weight DCA capsules (CBD capsule + DCA capsule). CBD capsules were combined with DCA capsules at the time of administration. The drug/s were mixed in raspberry jam, which improved palatability. The same amount of raspberry jam (mg) was given to all mice, including the naked CBD oil group to limit treatment variability. Prior to sacrifice, mice were anesthetised with isoflurane gas and blood was obtained via cardiac puncture into EDTA-coated tubes. Mice were euthanised by cervical dislocation and the brain was collected and snap frozen immediately at 0.3 hours, 1 hour, and 3 hours post-administration. Four mice were used per time point per group, except for the Capsule and Naked groups at 1 hour, where three mice were used. This was determined based on previous similar studies of CBD pharmacokinetics [9, 25]. Plasma was collected by centrifuge (2500 g, 10 minutes, 4°C). Samples were stored at -80°C until analyses.

### Measurement of CBD concentration

The concentration of CBD in plasma and brain tissue was determined using the protocol described previously [20]. Briefly, plasma samples were thawed and 50 μL were added to 100 μL of acetonitrile containing deuterated internal standards. Brain samples (500 mg) were homogenised in methanol (500 μL) using a Tissue-Lyser with a 3 mm steel ball bearing, the samples were then centrifuged and 50 μL of homogenate was added to 100 μL of acetonitrile containing deuterated internal standards. Both plasma and brain samples were vortexed, then centrifuged and the supernatant was transferred to a vial and injected onto the LCMS system (Shimadzu 8060, Shimadzu, Australia). A Kinetex Biphenyl column (50 ×3 mm, 2.6 μm) using a gradient of 0.1% formic acid and acetonitrile was used for the analysis. The calibration curve ranged from 0.5–500 ng/mL with a limit of quantitation of 0.5 ng/mL.

### Pharmacokinetic and statistical analysis

The results are expressed as mean and where applicable, the standard error of mean (SEM) is provided. Graphpad Prism v7 (Graphpad, Inc., USA) was used to generate concentration-time graphs on linearized log_10_ scale and calculate area under the concentration curve (AUC). Statistical significance between treatment groups was determined using one-way ANOVA with significance at *p* < 0.05.

Pharmacokinetic parameters were approximated using established models in Microsoft Excel using the add-in program PKSolver (Microsoft Excel 2010) and are expressed below as median [21]. These included terminal half-life (t_1/2_), maximum concentration (C_max_), time to reach Cmax (t_max_), area under the concentration curve from zero to a certain time (AUC_0–t_), and from zero to observed infinity (AUC _0-inf_obs_).

## Results and discussion

To improve the oral bioavailability of CBD, we assessed the pharmacokinetic profile of CBD capsules as a standalone treatment and in combination with capsules containing the permeation enhancer, DCA; compared to naked CBD oil. The microencapsulation technology utilised in this study was pioneered by our laboratory and has been adopted for enhanced lipophilic drug delivery in varied preclinical models of metabolic disorders [11, 15].

### Characterisation of capsulated CBD

The formulation that generated the most optimal, reproducible microcapsules was at 1.5% MVSA and 1% CBD. The microcapsules were spherical, and their size was 400±50 μm immediately post drying. The CBD capsules were white and their appearance remained consistent for 30 days at room temperature; followed by gradual discoloration with the capsules appearing brown, rough and shrived. Following the desiccation process, over 98% of capsulated CBD remained intact after exposure to the ambient air and light for 72 hours, indicating marked protection of CBD by the MVSA encapsulation. This data suggests that the encapsulation protocol may provide protection of CBD from exposure to ambient air, potentially improving shelf-life stability. Further tests are required to extensively characterise the physical properties and stability of the CBD capsules [22].

Calculated encapsulation efficiency of CBD was 23±1.2% and CBD loading was estimated as 2.05±0.10%. Drug loading was comparable to previous studies [23]. The encapsulation efficiency remained relatively low despite multiple optimisations. A recent study by Wagle et al. [23] that utilised a similar encapsulation protocol on probucol reported a drug loading of approximately 2% but with an encapsulation efficiency as high as 92%. The drug used in the aforementioned study was in powder form, which may have been a major determinant of the encapsulation efficiency. Further investigations will be needed to evaluate the effects of the oil content of our CBD preparation.

### Pharmacokinetics of capsulated CBD

The present study used a dosage of 5mk/kg, which is lower than the typically administered dosage of 20 mg/kg in previous studies [24, 25]. The small dosage was chosen to mitigate the low encapsulation efficiency, whilst yielding plasma and brain concentrations that could be detectable for this short-term study [26]. CBD pharmacokinetics, including C_max_ and AUC in plasma and brain samples have been demonstrated to be dose-dependent in both animal [27] and clinical studies [8], making it difficult to compare these results with published literature.

**Fig 1:**
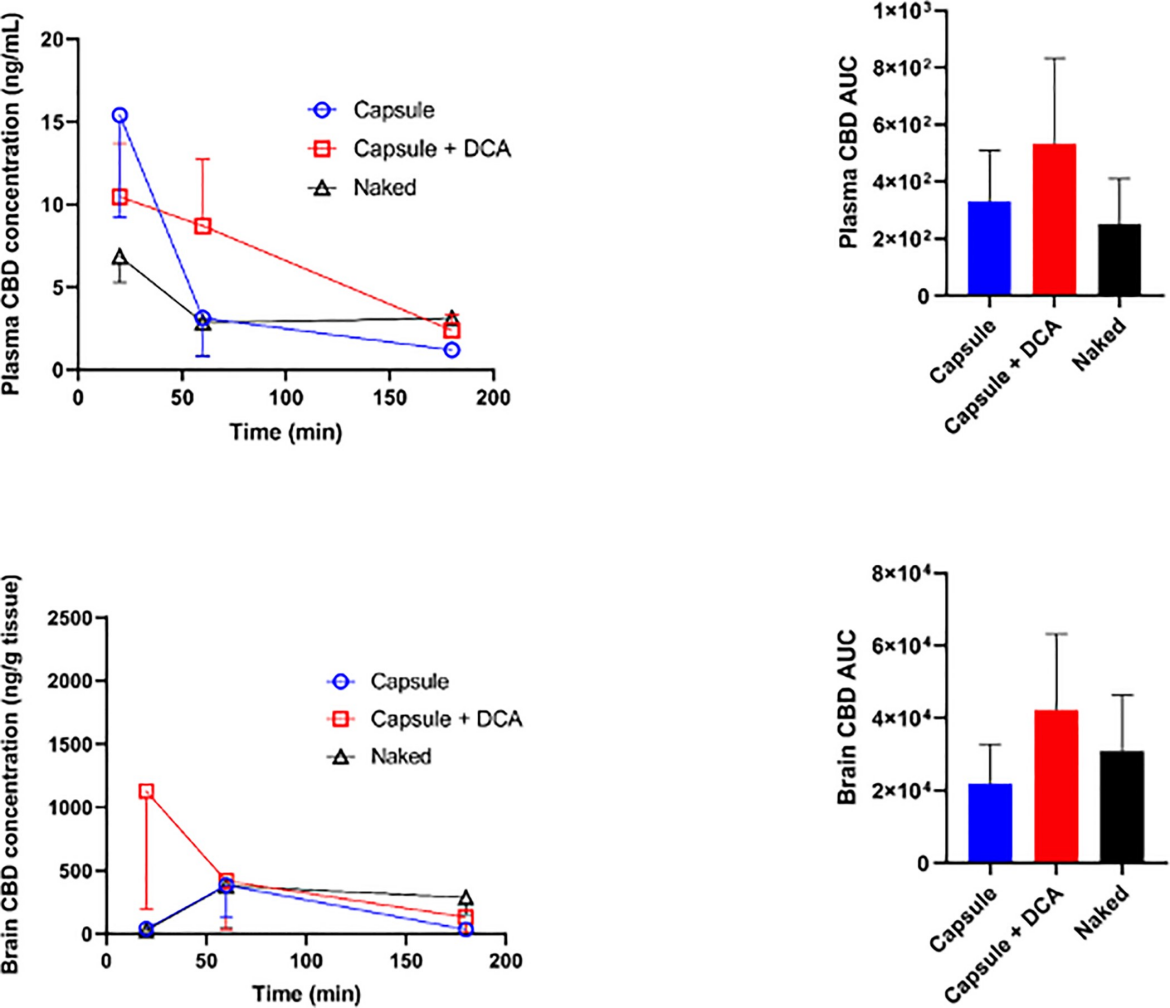
Mean concentration-time curve of Cannabidiol (CBD) in plasma and brain (a, c) with corresponding cumulative concentrations shown as area under curve (AUC) graphs (b, d). CBD was administered orally to mice at a dosage of 5 mg/kg according to the following formulations: 1) CBD capsules, 2) CBD capsules + deoxycholic acid (DCA) capsules (4 mg/kg) and 3) naked CBD oil. For each graph, the data represents Mean ±SEM bars (n = 3 or 4 per treatment group for each time point). Analysis using one-way ANOVA revealed no statistical significance, with plasma and brain data yielding *p* = 0.07 and *p* = 0.67, respectively.

Fig 1 illustrates the mean plasma and brain concentrations of CBD in mice administered with 1) CBD capsules, 2) CBD capsules + DCA capsules and 3) naked CBD oil–along with the corresponding area under curve (AUC) graphs. Data was accrued at three time points over 3 hours to focus on the acute bioavailability of CBD encapsulation. The short-term effects of CBD in mice are of interest to our lab due to their observed fast metabolic rates. For example, the plasma elimination half-life of injected CBD is shorter in mice at 4.5 hours [24] compared to humans at 24 hours [28]. Short-term pharmacokinetic studies, while not common, have been published with a focus on brain and plasma availability. A recent study looked at the penetrance of phytocannabinoid acids (CBDAs) in mice at 5 timepoints up to 2 hours post intraperitoneal injection [29]. Unsurprisingly, the study reported poor brain absorption of CBDAs, which limited the therapeutic outcomes of the drugs. This study aimed to optimise short-term pharmacokinetics to improve the neuroprotective effects of CBD.

At 0.3 hours post oral administration, the plasma levels of the CBD Capsule group was over 2-fold higher than the naked CBD oil group, whilst in the brain, the CBD concentrations of CBD capsule and naked oil groups were low, but comparable. When CBD capsules were given in combination with DCA capsules (CBD capsule + DCA capsule group), the mean brain concentration of CBD was 1048 ng/mg tissue, a remarkable 40- and 30-fold increase compared to the naked CBD oil and CBD capsule groups, respectively. The lower plasma CBD concentration was observed in CBD capsule + DCA capsule mice compared to the CBD capsule mice, possibly a consequence of enhanced tissue uptake. After 1 hour of oral administration, brain CBD concentration of CBD capsule + DCA capsule mice showed over a 60% reduction to 418 ng/mg, while CBD capsule group increased from 42 ng/mg to 385 ng/mg and naked CBD oil group increased from 28 ng/mg to 380 ng/mg. The latter groups showed increases of over 800% to reach peak concentrations, resulting in comparable brain CBD levels across all three treatment groups. The potential effect of DCA on the drastic reduction in brain CBD concentration is an interesting finding that requires further investigations. Research suggests that bile acids my promote cellular uptake and clearance of lipoproteins [16], however the effects of bile acids such as DCA on tissue clearance of lipophilic drugs such as CBD have yet to be defined.

In plasma, the CBD capsule group mice showed a reduction of CBD from 0.3 hours, whilst a moderate decrease was observed in the naked group, reaching similar plasma CBD concentrations. The plasma CBD concentration of CBD capsule + DCA capsule mice showed only a slight decrease and remained substantially higher than the CBD capsule or naked CBD oil group mice. In these CBD capsule + DCA capsule mice, plasma CBD continued to reduce toward the 3 hour time point, whereas the CBD capsule and naked CBD oil groups showed no decline or only a slight decline, resulting in similar plasma CBD levels across all three groups at the 3 hour time point. The brain concentrations of CBD in CBD capsule, CBD capsule + DCA capsule and naked CBD oil mice showed only a modest reduction from the 1 hour to 3 hour time points, indicating comparable brain CBD concentrations.

The AUC graphs on the right show the cumulative abundance over 3 hours post-oral administration. The plasma concentrations of CBD were comparable between the CBD capsule group and naked CBD oil group. The 3 hour cumulative plasma CBD was substantially higher in CBD capsule + DCA capsule mice, showing more than double the concentration of CBD capsule, or naked CBD oil group (Fig 1B). Similarly, in the brain, the 3 hour cumulative CBD concentration was markedly higher and was double in the CBD capsule + DCA capsule mice compared to the CBD capsule mice (Fig 1D).

The trends described did not reach statistical significance using ANOVA (plasma: *p* = 0.07 and brain: *p* = 0.67), however, the treatment effect may be obscured by the small sample sizes. Indeed, our previous work reported significantly increased absorption of a lipophilic drug with similarly produced MVSA microcapsules in a mouse study with n = 10 per treatment group [11].

The PK parameters generated using PKSolver in Microsoft Excel are summarised in Table 1. Plasma terminal half-life (t_1/2_) of CBD in mice that were given naked CBD oil was 2.2 h. The plasma CBD concentration in the naked CBD group mice reached its peak at 0.3 h post-oral administration, which was 7.7 ng/mL. The plasma absorption profile of CBD oil was comparable with published findings [25], albeit with minimal variation in the pharmacokinetic parameters that are likely to be dose dependent [8]. The current results demonstrate that plasma bioavailability as indicated by C_max_ and AUC_0-t_ was mildly increased for encapsulated CBD compared to naked oil (by 27.3% and 14.6%, respectively). With the same t_max_, we conclude that capsulation may have a similar rate of intestinal absorption as naked oil, however, it may protect against extensive first-pass metabolism to promote CBD into the surrounding capillaries.

**Table 1:** Pharmacokinetic parameters of Cannabidiol (CBD) administered orally to mice at a dosage of 5 mg/kg in the following formulations: 1) capsule, 2) CBD capsule + deoxycholic acid (DCA) capsule (4 mg/kg) and 3) naked CBD oil formulation, in plasma and brain.

		**Plasma**			**Brain**		
		**Capsule**	**Capsule + DCA**	**Naked**	**Capsule**	**Capsule + DCA**	**Naked**
**Parameter**	**Unit**						
t1/2	h	1.1	0.4	2.2	0.9	4.5	Not determined
t_max_	h	0.3	1.0	0.3	1.0	0.7	2.0
C_max_	ng/mL or ng/g	10.9	11.0	7.7	280.9	1048.2	421.5
AUC 0-t	ng/mL*h	15.2	16.7	12.9	368.1	1242.1	447.4
AUC 0-inf_obs	ng/mL*h	16.3	6.5	14.9	75.0	4378.4	Not determined

Parameters were generated using standard non-compartmental analysis in PKSolver with plasma and brain concentration data.

Each value is expressed as Median.

t_1/2_, terminal half-life; C_max_, maximum concentration; t_max_, time to reach C_max_; AUC0–t, area under the concentration curve from zero to 3 hours post oral administration; AUC _0-inf_obs_, AUC from zero to observed infinity.

The plasma t_1/2_ of CBD was halved for the CBD capsule group mice compared to naked CBD group mice, which alludes to increased clearance via excretion and/or tissue/cell uptake. The plasma t_1/2_ was shorter in CBD capsule + DCA capsule mice, indicating substantially enhanced CBD clearance from the plasma by the MVSA capsulation with DCA. Consistent with the latter, CBD analyses in brain tissue revealed in mice that were given CBD capsules, the t_max_ was 1 h, half of naked CBD group mice, indicating markedly faster brain tissue uptake of CBD by the MVSA capsulation. Furthermore, when the CBD capsules were administered with DCA in CBD capsule + DCA capsule group, the t_max_ was 1/3 of naked group mice, which also resulted in substantially higher C_max_, AUC 0-t and AUC _0-inf_obs_. These data indicate markedly increased brain tissue uptake of CBD by the MVSA encapsulation in combination with DCA. Moreover, the mice that were administered with CBD or CBD capsule + DCA capsule showed substantially longer t1/2, indicating prolonged retention of CBD within the brain.

The limitations of the present study include the low number of mice for each treatment group, which may have affected the statistical analysis and obscured potential treatment effects. Additionally, the study would have been strengthened by increasing the number of time-points for sample collections. Subsequent investigations could include 5 timepoints to clarify the short-term blood-to-brain kinetics of CBD. With further optimisation of the CBD encapsulation, along with *in vitro* characterisation of CBD capsules, future studies are likely to increase the drug dosage so results are more comparable with published data.

## Conclusion

Taken together, the outcomes of this study indicate that MVSA encapsulation may protect CBD from oxidation, degradation by light, and acidic digestion within the stomach, enhancing the absorption through the GI tract and cumulative plasma bioavailability. Although it was statistically non-significant, a bile acid, DCA, show increasing trend in uptake of CBD by up to 40 times within the brain and extends its retention. DCA may have a potential to promote the neuroprotective efficacy of orally administered CBD, particularly for the treatment of neurodegenerative disorders.

## References

[1] Iffland K, Grotenhermen F. An Update on Safety and Side Effects of Cannabidiol: A Review of Clinical Data and Relevant Animal Studies. Cannabis Cannabinoid Res. 2017; doi: 10.1089/can.2016.0034 28861514 PMC5569602

[2] Ibeas Bih C, Chen T, Nunn AVW, Bazelot M, Dallas M, Whalley BJ. Molecular Targets of Cannabidiol in Neurological Disorders. Neurotherapeutics. 2015. doi: 10.1007/s13311-015-0377-3 26264914 PMC4604182

[3] Martín-Moreno AM, Reigada D, Ramírez BG, Mechoulam R, Innamorato N, Cuadrado A, et al. Cannabidiol and other cannabinoids reduce microglial activation in vitro and in vivo: Relevance to alzheimer’s disease. Mol Pharmacol. 2011; doi: 10.1124/mol.111.071290 21350020 PMC3102548

[4] Rajesh M, Mukhopadhyay P, Bátkai S, Haskó G, Liaudet L, Drel VR, et al. Cannabidiol attenuates high glucose-induced endothelial cell inflammatory response and barrier disruption. Am J Physiol—Hear Circ Physiol. 2007; doi: 10.1152/ajpheart.00236.2007 17384130 PMC2228254

[5] Ruiz-Valdepeñas L, Martínez-Orgado JA, Benito C, Millán Á, Tolón RM, Romero J. Cannabidiol reduces lipopolysaccharide-induced vascular changes and inflammation in the mouse brain: An intravital microscopy study. J Neuroinflammation. 2011; 10.1186/1742-2094-8-5 PMC3034694 21244691

[6] Horvth B, Mukhopadhyay P, Hask G, Pacher P. The endocannabinoid system and plant-derived cannabinoids in diabetes and diabetic complications. American Journal of Pathology. 2012. 10.1016/j.ajpath.2011.11.003 PMC3349875 22155112

[7] Cabral GA, Jamerson M. Marijuana use and brain immune mechanisms. In: International Review of Neurobiology. 2014. doi: 10.1016/B978-0-12-801284-0.00008-7 25175866

[8] Millar SA, Stone NL, Yates AS, O’Sullivan SE. A systematic review on the pharmacokinetics of cannabidiol in humans. Frontiers in Pharmacology. 2018. doi: 10.3389/fphar.2018.01365 30534073 PMC6275223

[9] Bartner LR, McGrath S, Rao S, Hyatt LK, Wittenburg LA. Pharmacokinetics of cannabidiol administered by 3 delivery methods at 2 different dosages to healthy dogs. Can J Vet Res. 2018; 30026641 PMC6038832

[10] Rakesh P, Vipin K, Kanchan K. Alginate Beads Prepared by Ionotropic Gelation Technique: Formulation Design. Res J Chem Sci ISSN 2231-606X. 2015;

[11] Mamo JC, Lam V, Al-Salami H, Brook E, Mooranian A, Nesbit M, et al. Sodium alginate capsulation increased brain delivery of probucol and suppressed neuroinflammation and neurodegeneration. Ther Deliv. 2018; doi: 10.4155/tde-2018-0033 30277134

[12] Mooranian A, Raj Wagle S, Kovacevic B, Takechi R, Mamo J, Lam V, et al. Bile acid bio-nanoencapsulation improved drug targeted-delivery and pharmacological effects via cellular flux: 6-months diabetes preclinical study. Sci Rep. 2020; doi: 10.1038/s41598-019-53999-1 31919411 PMC6952395

[13] Wagle SR, Walker D, Kovacevic B, Gedawy A, Mikov M, Golocorbin-Kon S, et al. Micro-Nano formulation of bile-gut delivery: rheological, stability and cell survival, basal and maximum respiration studies. Sci Rep. 2020; doi: 10.1038/s41598-020-64355-z 32382021 PMC7205980

[14] Chiang JYL. Bile acid metabolism and signaling. Compr Physiol. 2013; doi: 10.1002/cphy.c120023 23897684 PMC4422175

[15] Mooranian A, Zamani N, Takechi R, Al-Sallami H, Mikov M, Goločorbin-Kon S, et al. Probucol-poly(meth)acrylate-bile acid nanoparticles increase IL-10, and primary bile acids in prediabetic mice. Ther Deliv. 2019; 10.4155/tde-2019-0052 31646943

[16] Pavlović N, Goločorbin-Kon S, Danić M, Stanimirov B, Al-Salami H, Stankov K, et al. Bile acids and their derivatives as potential modifiers of drug release and pharmacokinetic profiles. Front Pharmacol. 2018; doi: 10.3389/fphar.2018.01283 30467479 PMC6237018

[17] Mooranian A, Negrulj R, Chen-Tan N, Al-Sallami HS, Fang Z, Mukkur T, et al. Novel artificial cell microencapsulation of a complex gliclazide-deoxycholic bile acid formulation: A characterization study. Drug Des Devel Ther. 2014; doi: 10.2147/DDDT.S65396 25114507 PMC4122185

[18] Wagle SR, Kovacevic B, Walker D, Ionescu CM, Shah U, Stojanovic G, et al. Alginate-based drug oral targeting using bio-micro/nano encapsulation technologies. Expert Opinion on Drug Delivery. 2020. doi: 10.1080/17425247.2020.1789587 32597249

[19] Chan LW, Lim LT, Heng PWS. Microencapsulation of oils using sodium alginate. J Microencapsul. 2000; doi: 10.1080/02652040050161747 11063422

[20] P. G. Development of a simple LCMSMS method for THC and metabolites in plasma. Asia Pac J Clin Oncol. 2016;

[21] Zhang Y, Huo M, Zhou J, Xie S. PKSolver: An add-in program for pharmacokinetic and pharmacodynamic data analysis in Microsoft Excel. Comput Methods Programs Biomed. 2010; doi: 10.1016/j.cmpb.2010.01.007 20176408

[22] Kaur R, Kaur M, Singh J. Endothelial dysfunction and platelet hyperactivity in type 2 diabetes mellitus: molecular insights and therapeutic strategies. Cardiovasc Diabetol [Internet]. 2018;1–17. Available from: http://link.springer.com/10.1186/s12933-018-0763-3 30170601 10.1186/s12933-018-0763-3 PMC6117983

[23] Wagle SR, Kovacevic B, Walker D, Ionescu CM, Jones M, Stojanovic G, et al. Pharmacological and advanced cell respiration effects, enhanced by toxic human-bile nano-pharmaceuticals of probucol cell-targeting formulations. Pharmaceutics. 2020; doi: 10.3390/pharmaceutics12080708 32751051 PMC7463437

[24] Deiana S, Watanabe A, Yamasaki Y, Amada N, Arthur M, Fleming S, et al. Plasma and brain pharmacokinetic profile of cannabidiol (CBD), cannabidivarine (CBDV), Δ 9-tetrahydrocannabivarin (THCV) and cannabigerol (CBG) in rats and mice following oral and intraperitoneal administration and CBD action on obsessive-compulsive behaviour. Psychopharmacology (Berl). 2012; 10.1007/s00213-011-2415-0 21796370

[25] Xu C, Chang T, Du Y, Yu C, Tan X, Li X. Pharmacokinetics of oral and intravenous cannabidiol and its antidepressant-like effects in chronic mild stress mouse model. Environ Toxicol Pharmacol. 2019; doi: 10.1016/j.etap.2019.103202 31173966

[26] Malfait AM, Gallily R, Sumariwalla PF, Malik AS, Andreakos E, Mechoulam R, et al. The nonpsychoactive cannabis constituent cannabidiol is an oral anti-arthritic therapeutic in murine collagen-induced arthritis. Proc Natl Acad Sci U S A. 2000; doi: 10.1073/pnas.160105897 10920191 PMC16904

[27] Long LE, Chesworth R, Huang XF, Wong A, Spiro A, McGregor IS, et al. Distinct neurobehavioural effects of cannabidiol in transmembrane domain neuregulin 1 mutant mice. PLoS One. 2012; doi: 10.1371/journal.pone.0034129 22509273 PMC3317922

[28] Bruni N, Pepa C Della, Oliaro-Bosso S, Pessione E, Gastaldi D, Dosio F. Cannabinoid delivery systems for pain and inflammation treatment. Molecules. 2018. doi: 10.3390/molecules23102478 30262735 PMC6222489

[29] Anderson LL, Low IK, Banister SD, McGregor IS, Arnold JC. Pharmacokinetics of Phytocannabinoid Acids and Anticonvulsant Effect of Cannabidiolic Acid in a Mouse Model of Dravet Syndrome. J Nat Prod. 2019 10.1021/acs.jnatprod.9b00600 31686510

